# The role of flavonoid antioxidant, morin in improving procarbazine-induced oxidative stress on testicular function in rat

**DOI:** 10.1016/j.pbj.0000000000000028

**Published:** 2018-08-03

**Authors:** Ebenezer Tunde Olayinka, Ayokanmi Ore, Oluwatobi Adewumi Adeyemo, Olaniyi Solomon Ola

**Affiliations:** Biochemistry Division, Department of Chemical Sciences, Faculty of Natural Sciences, Ajayi Crowther University, Oyo, Nigeria.

**Keywords:** morin, oxidative stress, procarbazine, rat, testicular function

## Abstract

**Background:**

Procarbazine (PCZ) is an effective chemotherapeutic drug used in the treatment of lymphoma; however, oxidative stress–mediated testicular toxicity is a major side effect. Recently, therapeutic intervention using flavonoids against oxidative stress–related pathologies is gaining more attention. Morin (MOR) is a natural flavonoid with proven antioxidant activity. This study was designed therefore to evaluate the potential role of MOR in ameliorating PCZ-induced testicular oxidative stress and altered sperm quality in rat model.

**Methods:**

A total of 24 male Wistar rats (170–180 g) were randomly assigned into 4 treatment groups: I, control; II, PCZ (2 mg/kg b.w.); III, PCZ (2 mg/kg b.w.) + MOR (100 mg/kg b.w.) simultaneously administered and IV, MOR (100 mg/kg b.w.), and all treatments lasted 14 days.

**Results:**

PCZ treatment displayed significant reduction in sperm number, sperm motility, percentage normal sperm cells, and daily sperm production rate. Meanwhile the activities of testicular enzymes: gamma-glutamyl transferase, acid phosphatase, and lactate dehydrogenase were significantly altered in the PCZ group compared to control. Furthermore, PCZ caused a significant reduction in levels of glutathione and ascorbic acid as well as activities superoxide dismutase, catalase, glutathione peroxidase, and glutathione S-transferase in the testes of PCZ-treated rats. A significant increase in testicular malondialdehyde level was also observed in the PCZ group. MOR treatment, however, significantly restored the altered sperm parameters and biochemical markers in the testis.

**Conclusions:**

Our data suggest that MOR administration protected against PCZ-induced testicular and spermatotoxicity in rat, by improving testicular antioxidant system.

## Introduction

In recent years, chemotherapy has improved the survival rates in many cancer cases such as acute lymphoblastic leukemia, Hodgkin disease, and testicular tumor.^1^ Chemotherapeutic applications has, however, resulted in a number of acute and chronic organ toxicities.^2^ In most cases, the effectiveness of some of these chemotherapeutic agents has been limited by these side effects.^3^ Among the side effects of concern is chemotherapy-induced male infertility.^4,5^ This progresses with gradual loss of testicular function and is often associated with oxidative stress, azoospermia, alteration in testicular morphology, and spermatogenesis.^6,7^ An important class of chemotherapeutic drugs, the alkylating agents, are capable of causing male infertility as reported with the frequently used curative regimens, chlorambucil, cyclophosphamide, and procarbazine (PCZ).^7–9^

**Figure 1:**
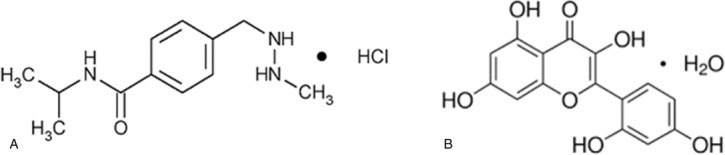
Chemical structures of procarbazine (A) and morin (B).

PCZ [*N*-isopropyl-a-(2-methyl-hydrazine)-*p*-toluamide hydrochloride] (Fig. 1A) is an orally administered alkylating agent employed for the treatment of Hodgkin lymphoma, malignant melanoma, and primary central nervous system lymphoma.^10^ PCZ induces organ toxicity through generation of free radicals (FRs) and disruption of physiological antioxidant defense system.^11,12^ It is known to cause testicular damage at a high rate, even after a single dose.^9,13^ Testicular damage from PCZ is characterized by failure of spermatogenesis and this represents a major limiting factor for the use of the drug.^14,15^

The level of the nonenzymatic antioxidants, glutathione (GSH), and ascorbic acid (AA) is vital to testicular function as a primary antioxidant defence. AA contributes to spermatogenesis by stimulating sperm production and testosterone secretion. It maintains testicular α-tocopherol in an active state. AA itself is maintained in a reduced state by a GSH-dependent dehydroascorbate reductase, which is abundant in the testes.^16^

Most often, organ systems recover from toxicities after cessation of chemotherapy, due to a rapid cellular regeneration and repairs.^17^ Moreover, the high testicular content of highly unsaturated fatty acids may predispose it to FR-induced lipid peroxidation.^16^ It, therefore, follows that preventive measures against FR-induced testicular damage may offer protection against testicular injury and male infertility experienced by patients receiving these chemotherapeutic agents. A potential solution lies in the investigation of protective substances which could eventually offer significant clinical advantage in man.^14^ One such substance is the flavonoid morin (MOR; 3,5,7,20,40-pentahydroxyflavone, Fig. 1B) a member of flavonols, found in Morus alba L (white mulberry) and red Wine, almond (*Prunus dulcis*, family Rosaceae), in sweet chestnut (*Castanea sativa*, family Fagaceae) *Acridocarpus orientalis*, and other fruits also.^18,19^ MOR was shown to have several pharmacological benefits including FR scavenging activity, anticancer activity, anti-inflammatory activity, protection against DNA damage, and prevention of low-density lipoprotein oxidation among others.^19^ Considering the antioxidant properties of MOR, it is thought that coadministration with PCZ may offer protection against the FR-mediated alteration in testicular function induced by PCZ. The present study was therefore designed to evaluate the ameliorative effects of MOR against PCZ-induced testicular oxidative stress and alteration in sperm parameters in rat model.

## Materials and methods

### Chemicals and reagents

The following substances were employed in the study: PCZ hydrochloride (Naprod Life Sciences Pvt Ltd, Mumbai, India); MOR hydrate, thiobarbituric acid (TBA), GSH, 1-chloro-2,4-dinitrobenzene, epinephrine, 5’,5’-dithio-bis-2-nitrobenzoic acid, para-nitrophenyl phosphate, and hydrogen peroxide (Sigma Chemical Company, London, UK). Assay kits for gamma-glutamyl transferase (γ-GT), lactate dehydrogenase (LDH) (Randox Laboratories Ltd, Antrim, UK). All other substances used were of analytical grade.

### Animal selection and care

A total of 24 adult male Wistar rats (170–180 g) were used in this study. They were obtained from the animal housing unit, Department of Chemical Sciences, Ajayi Crowther University, Oyo, Nigeria. The rats were acclimatized to laboratory conditions for 2 weeks before the commencement of study. The rats were housed in wire-meshed cages and supplied food and water ad libitum. Handling of the experimental animals follows international guidelines on animal use and reporting.^20,21^ The animal experiment was approved by the Faculty of Natural Sciences Ethical Review Committee (FNS/ERC/201700006).

### Experimental design

Animals were assigned into 4 treatment groups (n = 6/group): I control (distilled water p.o.); II [2 mg/kg body weight (b.w.) PCZ p.o.]; III (2 mg/kg b.w. PCZ + 100 mg/kg b.w. MOR p.o. administered simultaneously); and IV (100 mg/kg b.w. MOR p.o.) The dose for PCZ (2 mg/kg b.w.) was selected based on the recommended adult dose for Hodgkin diseases, and a previous work in our laboratory,^12^ whereas the dose for MOR (100 mg/kg b.w.) was chosen based on available literature.^22^ Respective dose were delivered in 1 mL of distilled water orally, once daily by oral intubation. All treatments lasted for 14 days^12^ and animals were euthanized 24 hours after the final treatments. Laparotomy was conducted and testes (with the epididymis) were removed from each animal and immediately used for sperm analysis.

### Determination of testicular and epididymal sperm number, progressive sperm motility, and volume

Epididymal and testicular sperm was obtained by mincing the caudal epididymis and the testis in normal saline and filtering through a nylon mesh. Sperm cells were counted using the Neubauer hemocytometer as previously described.^23^ The motility of epididymal sperm was evaluated visually at ×400 magnification within 2 to 4 minutes of their isolation from the cauda. Motility estimations were performed from the entire field in each sample. The mean was used as the final motility score and data were expressed as percentages.^24^

### Morphological examination of spermatozoa

A portion of the sperm suspension placed on a glass slide was smeared out with another slide, fixed in 95% ethanol, and stained with 1% eosin and 5% nigrosin for morphological and viability observation. At least 100 sperms from each rat were examined for abnormalities in different regions of spermatozoa according to the method described by Wyrobek et al.^25^

### Daily sperm production rate

The testis was weighed, decapsulated, and homogenized in ice-cold 0.9% sodium chloride. The homogenate was filtered through a nylon mesh to remove connective tissue, and the filtrate was used to count the number of homogenization-resistant spermatids/sperm in each sample in duplicate using a hemocytometer. Calculation of daily sperm production (DSP) was done by dividing the total number of spermatids/sperm per gram testis by 6.1 days (the duration of step 19 spermatids in the seminiferous tubules).^26^

### Preparation of subcellular fraction of testicular samples

Each testis was rinsed in ice-cold 1.15% KCl, blotted of blood stains and weighed. Each testis was homogenized in 9 volumes of ice-cold 0.1 M phosphate buffer (pH 7.4) (1:10). The homogenates were subjected to centrifugation at 12,000 × *g* for 10 minutes in refrigerated centrifuge maintained at 4°C (Eppendorf Ltd, Stevenage, UK). The supernatant obtained was carefully collected in sample tubes and stored frozen for subsequent biochemical assays.

### Biomarkers of testicular function

Activities of testicular γ-GT, acid phosphatase (ACP), and LDH were assayed using assay kits (RANDOX) following the manufacturer's procedure. γ-GT activity was determined by the principle described by Szasz.^27^ ACP activity was determined by the method of Tietz.^28^ LDH activity was determined based on the method of Cabaud and Wroblewski.^29^

### Testicular markers of oxidative stress

#### Reduced glutathione

Reduced GSH level was determined according to Jollow et al.^30^ Reduced GSH reacts with Ellman's reagent to give a chromophoric product, 2-nitro-5-thiobenzoic acid with a molar absorption at 412 nm. Briefly, the reaction mixture consists of 0.2 mL of testicular homogenate, 1.8 mL of dH_2_O, and 3 mL of sulfosalicylic acid (4%). The mixture was allowed to stand for 5 minutes and then filtered. One milliliter of the filtrate was added to 4 mL of phosphate buffer (0.1 M) and 0.5 mL of 0.04% Ellman's reagent prepared in 0.1 M phosphate buffer (pH 7.4). A blank was constituted with 4 mL of the 0.1 M phosphate buffer, 1 mL of sulfosalicylic acid, and 0.5 mL of the Ellman's reagent. The absorbance was measured at a wavelength of 412 nm. Reduced GSH concentration in the testicular homogenate was extrapolated from the standard curve for GSH.

### Ascorbic acid

AA level was determined following the procedure of Jagota and Dani.^31^ Folin Ciocalteu (Folin-phenol) reagent, reacts with AA in biological samples to give a blue color, which absorbs maximally at 760 nm. Approximately 0.5 mL of testicular fraction was added to 0.8 mL of trichloroacetic acid in a test tube, followed by vigorous shaking. After the tubes were cooled in an ice for 5 minutes, they were subjected to centrifugation at 3000 × *g* for 5 minutes. Two milliliters of supernatants obtained were reacted with 0.2 mL of Folin's reagent (diluted 10-fold in distilled water) and stirred vigorously. After 10 minutes, the absorbance of the blue chromophore developed was measured at 760 nm. AA (μg/mL) in the testicular fraction was obtained from the standard curve for AA.

### Glutathione peroxidase

Activity of glutathione peroxidase (GPx) was determined by the procedure of Rotruck et al.^32^ To 0.5 mL of phosphate buffer (0.1 M, pH 7.4) in a test tube was added 0.1 mL of 10 mM NaN_3_, 0.2 mL of GSH (4 mM), 0.1 mL of 2.5 mM H_2_O_2_, and 0.5 mL of testicular fraction. The reaction mixture was incubated for at 37°C for 3 minutes after which 0.5 mL of 10% trichloroacetic acid was added. The resulting mixture was centrifuged at 3000 rpm for 5 minutes. One milliliter of the supernatant obtained was reacted with 2 mL of 0.3 M K_2_HPO_4_ and 1 mL of 5’,5’-dithio-bis-2-nitrobenzoic acid and the absorbance read against a reagent blank at 412 nm. GPx activity is expressed as μg GSH/mg protein.

### Catalase

Catalase (CAT) activity was determined by the method described by Sinha^33^ based on the reduction of dichromate in acetic acid to chromic acetate when heated in the presence of hydrogen peroxide (H_2_O_2_). The assay mixture, 4 mL of H_2_O_2_ solution (800 μmol), 5 mL of phosphate buffer (0.01 M, pH 7.0), 1 mL of diluted testicular post mitochondrial fraction (PMF) (1:50) was rapidly mixed at room temperature. A 1 mL portion of reaction mixture was withdrawn and blown into 2 mL dichromate/acetic acid reagent at 60 seconds intervals to determine the amount of H_2_O_2_ remaining. The chromic acetate produced was measured spectrophotometrically at 570 nm and the amount of H_2_O_2_ remaining was extrapolated from the standard curve for H_2_O_2_. CAT activity was expressed as micromole of H_2_O_2_ consumed per minute per mg protein.

### Glutathione S-transferase

Glutathione S-transferase (GST) activity was determined by the method described by Habig et al.^34^ Briefly, the reaction mixture (3 mL) was made up of 30 μL of reduced GSH (0.1 M), 150 μL of 1-chloro-2,4-dinitrobenzene (3.37 mg/mL), 2.79 mL phosphate buffer (0.1 M, pH 6.5), and 30 μL of liver PMF. The reaction was allowed to run for 60 seconds before the absorbance was measured at 340 nm against the blank. GST activity in the testis was expressed in micromole GSH consumed per minute per milligram protein.

### Superoxide dismutase

Activity of superoxide dismutase (SOD) in the testis was measured according to Misra and Fridovich^35^ by measuring the inhibition of auto-oxidation of epinephrine under alkaline condition. One milliliter of the sample was diluted in 9 mL of distilled water to obtain a 1 in 10 dilution. An aliquot of 0.2 mL of the diluted enzyme preparation was added to 2.5 mL of 0.05 M carbonate buffer (pH 10.2), equilibrated in the spectrophotometer, and the reaction was started by the addition of 0.3 mL of freshly prepared 0.3 mM epinephrine to the mixture which was quickly mixed by inversion. The reference cuvette contained 2.5 mL of carbonate buffer, 0.3 mL of adrenaline, and 0.2 mL of distilled water. The increase in absorbance at 480 nm was monitored every 30 seconds for 150 seconds. SOD activity is expressed in unit per milligram of protein. One unit of SOD activity is defined as the amount of SOD necessary to cause 50% inhibition of the oxidation of adrenaline to adrenochrome over an interval of 1 minute.

### Lipid peroxidation

Level of lipid peroxidation (LPO) was determined according to the method of Varshney and Kale.^36^ The method involved the reaction between malondialdehyde (MDA; product of lipid peroxidation) and TBA to yield a stable pink chromophore with maximum absorption at 532 nm. Briefly, the reaction mixture consisted of 1.6 mL Tris-KCl buffer, 0.4 mL of the test sample, 0.5 mL of 30% TCA, and 0.5 mL of 0.75% TBA and the mixture was placed in a water bath for 1 hour at 95°C. This was then cooled and subjected to centrifugation at 3000 rpm. The supernatant was collected and the absorbance measured against a blank of distilled water at 532 nm.

### Statistical analysis

Data are expressed as the mean ± standard (SD) for 6 rats. Statistical analysis and graphical constructions were performed using Graphpad Prism 6.0.1 (Graphpad Software, La Jolla, CA). The statistical significance of differences between experimental groups were determined by 1-way analysis of variance and complemented with Tukey multiple comparison test. *P* values of <.05 were considered to be significant.

## Results

### Influence of MOR on procarbazine-induced changes in sperm parameters in rats

**Figure 2:**
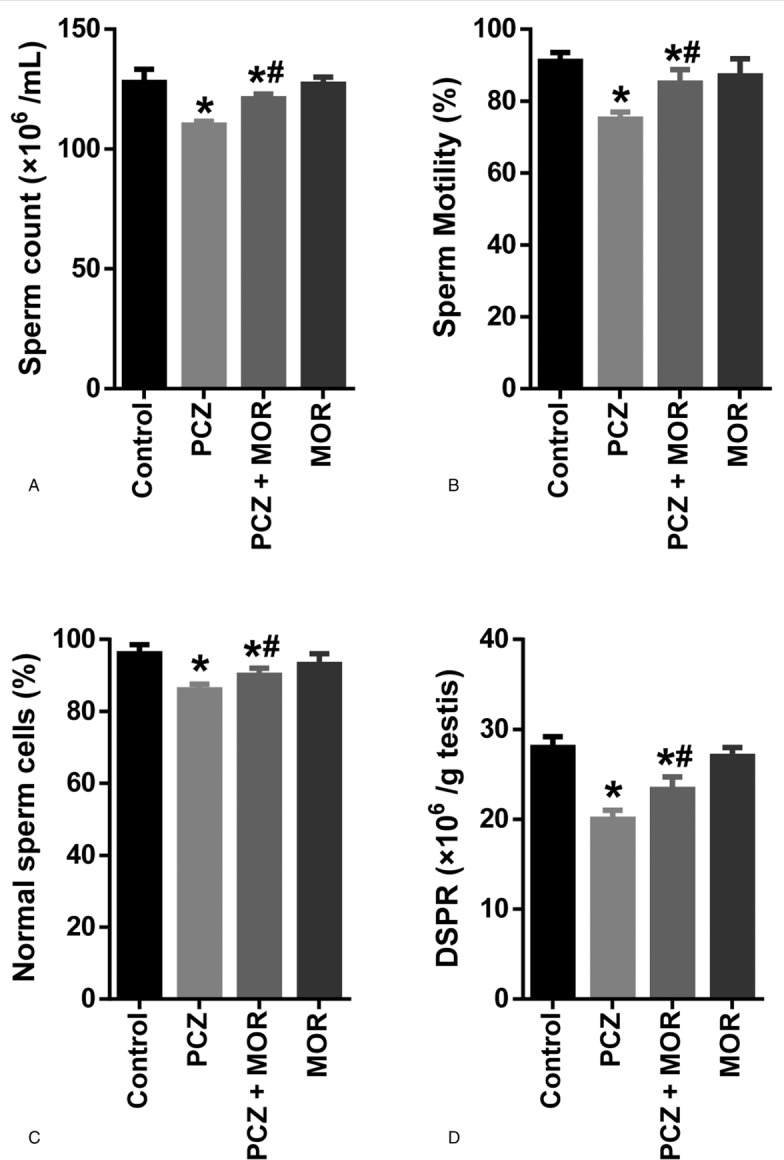
Ameliorative effect of MOR on PCZ-induced changes in sperm parameters in rats: sperm count (A); sperm motility (B); percentage normal sperm cells (C); and daily sperm production rate (D). Each bar represents the mean ± standard deviation of 6 rats. ^∗^ Significantly different compared with control; ^#^ Significantly different compared with PCZ (*P* *<* .05). DSPR = daily sperm production rate; MOR = morin; PCZ = procarbazine.

Figure 2 represents the protective effect of MOR on PCZ-induced changes in sperm parameters. PCZ caused a significant (*P* *<* .05) reduction in number sperm cells as shown in Figure 2A. Similar reduction was also observed in percentage sperm cell motility and normal sperm cells (Fig. 2B and C, respectively). Furthermore, DSP rate also declined significantly after administration of PCZ (Fig. 2D). However, administration of MOR significantly ameliorated the alterations in sperm parameters induced by PCZ.

### Influence of MOR on procarbazine-induced changes in testicular function indices in rats

**Figure 3:**
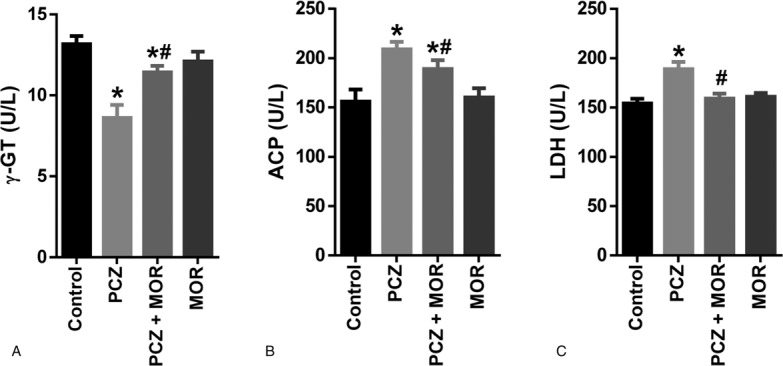
Ameliorative effect of MOR on PCZ-induced alterations in testicular function biomarkers in rats: γ-GT (A); ACP (B); and LDH (C). Each bar represents the mean ± standard deviation of 6 rats. ^∗^ Significantly different compared with control; ^#^ Significantly different compared with PCZ (*P* *<* .05). ACP = acid phosphatase; γ-GT = gamma-glutamyl transferase; LDH = lactate dehydrogenase; MOR = morin; PCZ = procarbazine.

To further understand the protective role of MOR in PCZ-induced testicular toxicity, we also determined the activities of selected testicular enzymes: γ-GT, ACP, and LDH (Fig. 3). PCZ caused a significant reduction in testicular activity of γ-GT. Conversely, a significant increase was observed in the activities of ACP and LDH in PCZ-treated animals compared to control. However coadministration with MOR ameliorated the observed PCZ-induced alterations in the activities of these enzymes.

### Influence of MOR on procarbazine-induced changes in testicular biomarkers of oxidative stress in rats

**Figure 4:**
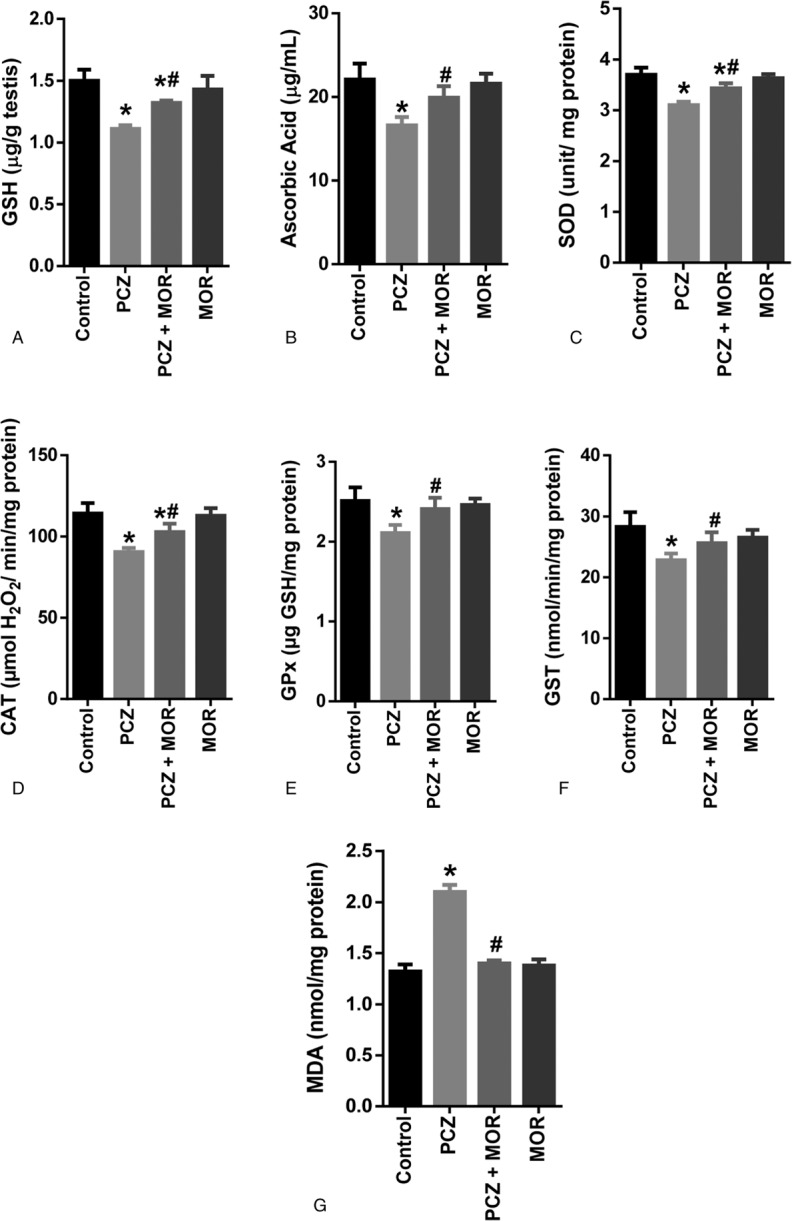
Ameliorative effect of MOR on PCZ-induced alterations in testicular biomarkers of oxidative stress in rats: reducedGSH (A); AA (B); SOD (C); CAT (D); GPx (E); GST (F); and MDA (G). Each bar represents the mean ± standard deviation of 6 rats. ^∗^ Significantly different compared with control; ^#^ Significantly different compared with PCZ (*P* *<* .05). AA = ascorbic acid; CAT = catalase; GPx = glutathione peroxidase; GSH = glutathione; GST = glutathione S-transferase; MDA = malondialdehyde; MOR = morin; PCZ = procarbazine; SOD = superoxide dismutase.

Testicular levels of the nonenzymatic antioxidants: reduced GSH and AA were significantly reduced (*P* *<* .05) after administration of PCZ (Fig. 4A and B, respectively). A similar reduction was also observed in the testicular activities of enzymatic antioxidants: SOD, CAT, GPx, and GST (Fig. 4C, D, E, and F, respectively). However, cotreatment with MOR significantly ameliorated the levels of GSH and AA and the activities of testicular SOD, CAT, GPx, and GST in rats.

MDA is a product of lipid peroxidation resulting from oxidative stress. Compared to the control group, PCZ induced a significant (*P* *<* .05) increase in the testicular LPO (as indicated by the MDA content in Fig. 4G). Cotreatment with MOR, however, attenuated this increase in testicular MDA levels.

## Discussion

One of the most implicated class of chemotherapy in male reproductive impairment is the alkylating agents. PCZ is a well-known alkylating agent causing sterility even within the first few doses of chemotherapy.^37^ PCZ is an effective chemotherapeutic drug used in lymphoma treatment with attendant testicular toxicity as a common side effect. Consequently, various ways of treatments (combining hormones and antioxidant) are being investigated to preserve testicular function during drug use.^38,39^ A mechanism of spermatotoxicity induced by PCZ is via bioactivation of PCZ to reactive intermediate along with formation of FRs during its metabolism.^13^

The present study investigated the potential role of the flavonoid antioxidant, MOR in the prevention of PCZ-induced testicular and spermatotoxicity in rats. To achieve this, rats were simultaneously administered PCZ and MOR. Evaluation of sperm parameters (total sperm count, motility, morphology, and DSP) constitutes an important marker of male reproductive function in cases of exposures to chemotherapeutic agents.^40,41^ Sperm motility is the sperm's ability to move while morphology refers to characteristic appearance of the head, neck, and tail.^42^ The observed reduction in the sperm function parameters is characteristic of spermatotoxicity induced by PCZ and other alkylating agents as previously reported.^8,43^ The ameliorative effect displayed by MOR against PCZ-induced alterations in sperm parameters is similar to reports from previous studies.^44,45^

A reduction in testicular γ-GT activity was observed after PCZ treatment in rats. γ-GT is involved in the metabolism of extracellular GSH and it is a useful biomarker of sertoli cell function.^46^ The observed decrease in γ-GT activity in the testes of PCZ-treated animals may have negative influence on the delivery of GSH to testicular cells.^47^ Testicular LDH activity is primarily associated with energy metabolism and it is useful marker for spermatogenic cell function and drug-induced testicular toxicity.^48^ The PCZ-induced increase in testicular LDH activity may impair spermatogenic energy metabolism and consequently mobility.^49^ ACP activity is present in lysosomes of Leydig cells and is involved in the removal of unrequired sperm cells. Data from the present study showed a significant increase in ACP activity after PCZ treatment in rats. This may reflect an increased rate in lytic activity in the testis due to increase requirement to eliminate superfluous sperm cells.^50^

Current data showed that PCZ caused a significant alteration in the testicular antioxidant status. This PCZ-induced oxidative stress is similar to that of doxorubicin in a recent study by Magalhães et al.^51^ FR-induced oxidative stress contributes to the formation of abnormal sperm and decreased sperm count and may have fatal effects on sperm function and fertility.^52^ Therefore, the RS produced in the testis and sperm must be inactivated continuously to prevent oxidative damage to sperm cells and preserve fertility potential.^53^

A decrease in levels of GSH and AA as well as the activities of testicular SOD, CAT, GPx, and GST was observed. GSH is a tripeptide involved in a variety of metabolic processes, including amino acid transport across membranes, detoxification of xenobiotics, maintenance of GSH levels in proteins and removal of FRs. GSH has been suggested to play a vital role in germ cell development.^54^ AA and GSH play an important role as FR scavengers in cells and protection against oxidation in tissues.^55,56^ Activities of SOD, CAT, GPx, and GST are essential in the maintenance of redox balance in the testis. Testicular SOD is involved in the dismutation of the harmful superoxide radical to H_2_O_2_ and O_2_,^57^ whereas CAT and GPx transform H_2_O_2_ into H_2_O and oxygen. GST on the contrary is involved in the detoxification of xenobiotics using GSH as substrate. PCZ-induced significant reduction in testicular levels of GSH and AA as well as activities of SOD, CAT, GPX, and GST, leading to increase in LPO. However, administration of MOR significantly ameliorated the PCZ-induced oxidative stress and lipid peroxidation, which agrees with previous reports on the antioxidant protection by MOR against oxidative stress caused by alkylating agents and other RS.^44^

## Conclusion

Combination chemotherapy is rapidly becoming a standard for treatment of patients with cancers. Combinations that include antioxidants may offer advantages in terms of survival and cure rates. Our study indicates that MOR exerts protection against PCZ-induced testicular toxicity, spermatotoxicity, and oxidative stress in rats. This may be attributed to its antioxidant activity. Therefore, MOR may find therapeutic potential in conditions in which chemotherapeutic agents pose damage to male reproductive system as a consequence of oxidative stress. However, further studies is required to elucidate the exact mechanism of action of MOR against PCZ toxicity and any potential interaction with the cytotoxic action of the drug.

## Conflicts of interest

The author(s) declared no potential conflicts of interest with respect to the research, authorship, and/or publication of this article.
